# Supplementary material for: Ion-Specific Gelation and Internal Dynamics of Nanocellulose Biocompatible Hybrid Hydrogels: Insights from Fluctuation Analysis
Source: Gels. 2025 Mar 12;11(3):197. doi: 10.3390/gels11030197 (PMC11942523; doi:10.3390/gels11030197)
Supplement: Supplementary file 1 [file gels-11-00197-s001.zip › gels-3502042-ESI-main.pdf]

Electronic Supplementary Information
Ion-Specific Gelation and Internal Dynamics of Nanocellulose Biocompatible Hybrid Hydrogels: Insights from Fluctuation Analysis

Arianna Bartolomei[1], Elvira D'Amato[1], Marina Scarpa[1], Greta Bergamaschi[2], Alessandro Gori[2], and Paolo Bettotti[1,*]

[1]Nanoscience Laboratory, Department of Physics, University of Trento, v. Sommarive 14, 38123 Povo, Trento, Italy.

[2]National Research Council of Italy, Istituto di Chimica Del Riconoscimento Molecolare (ICRM), Via Mario Bianco, 9, Milano, 20131, Italy

[*]Corresponding author: paolo.bettotti@unitn.it

1. Linear Viscoelastic Regions.

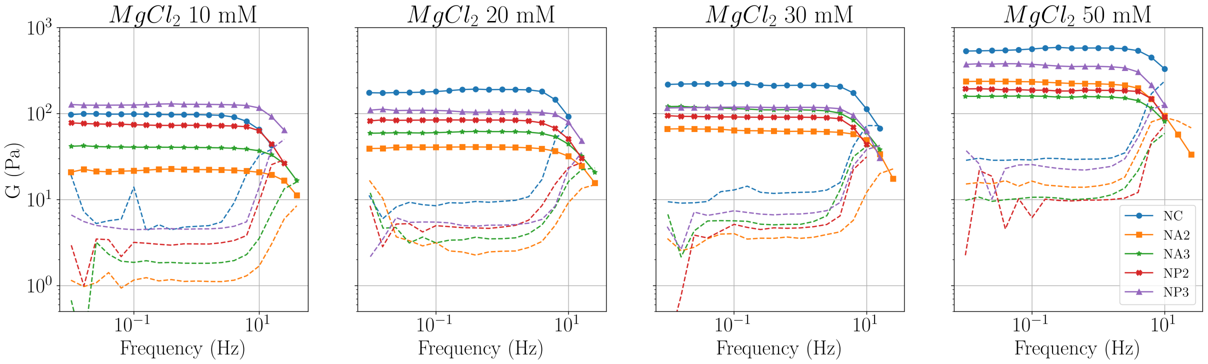

Figure 1: Linear Viscoelastic Region for each hydrogel composition investigated. Solid line with markers reports the Elastic moduli, dotted lines report the Viscous moduli. Please refers to the article main text for the different sample compositions listed in the legend.

2. Frequency Sweeps.

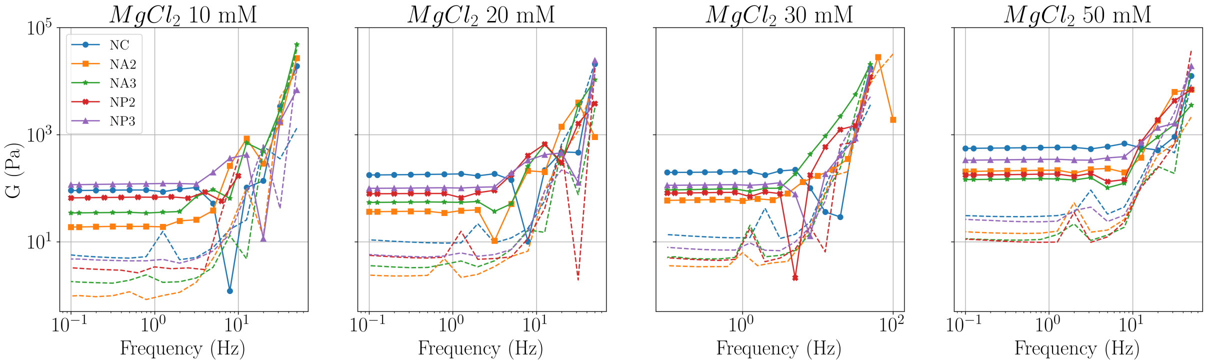

Figure 2: Frequency Sweep analysis for each hydrogel composition investigated. Solid line with markers reports the Elastic moduli, dotted lines report the Viscous moduli. Please refers to the article main text for the different sample compositons listed in the legend.

3. σ^2 and Static Light Scattering.

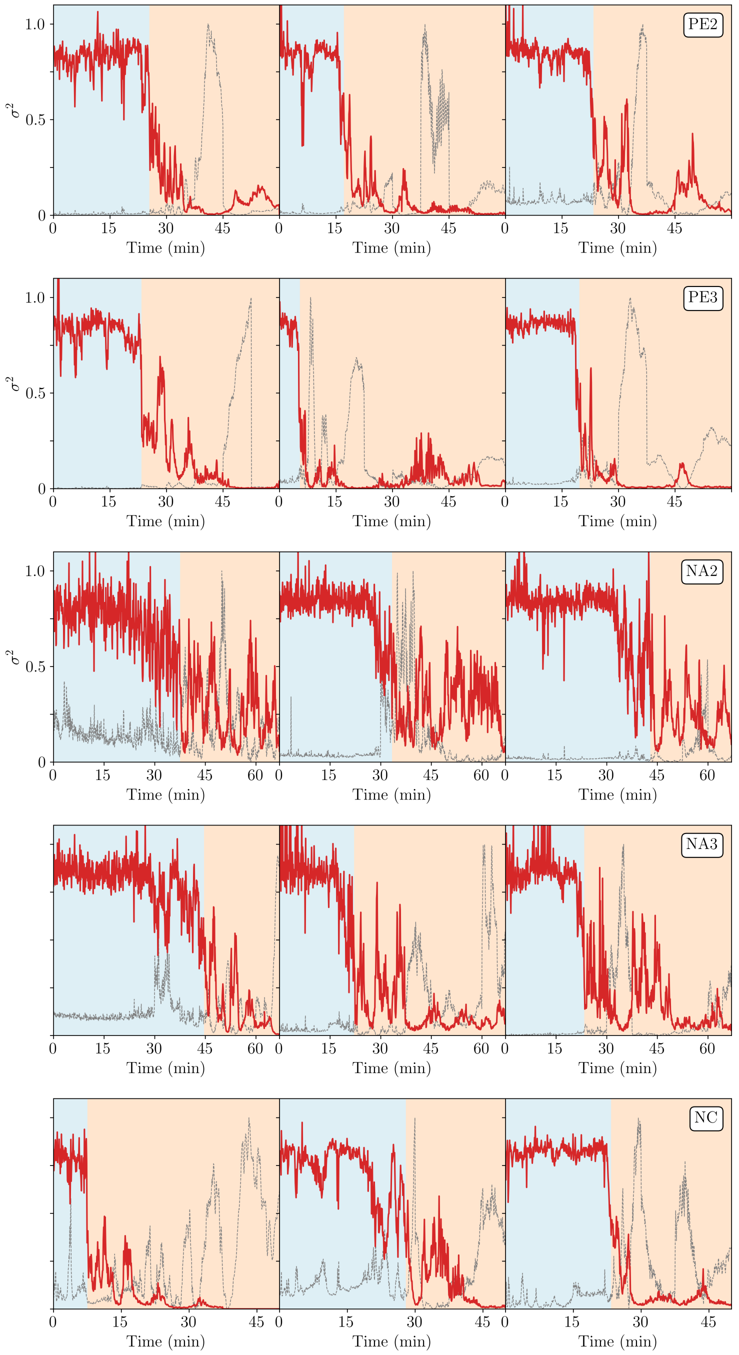

Figure 3: σ^2 and Static Light Scattering for all compositions investigated. Labels indicate the composition for the specific subplot row. The red line indicates σ_2^2, the grey line is the time average of the static light scattered during each run, normalized to unit range (time duration of each runs: 5 s).
