# Supplementary figures and images for: Ion-Specific Gelation and Internal Dynamics of Nanocellulose Biocompatible Hybrid Hydrogels: Insights from Fluctuation Analysis
Source: Gels. 2025 Mar 12;11(3):197. doi: 10.3390/gels11030197 (PMC11942523; doi:10.3390/gels11030197)

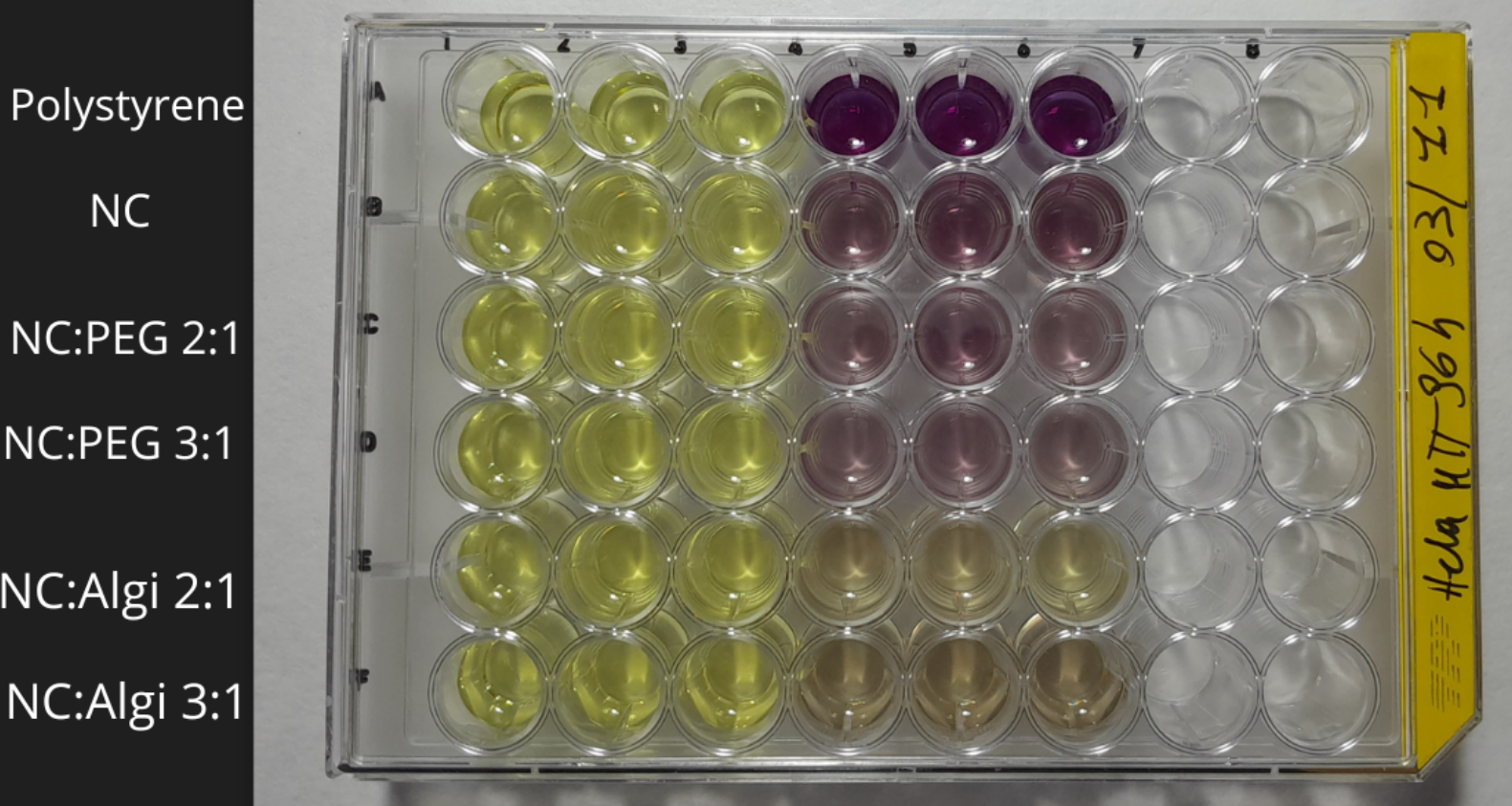

Supplement: Supplementary file 1 [file gels-11-00197-s001.zip › cell_cultures.png]

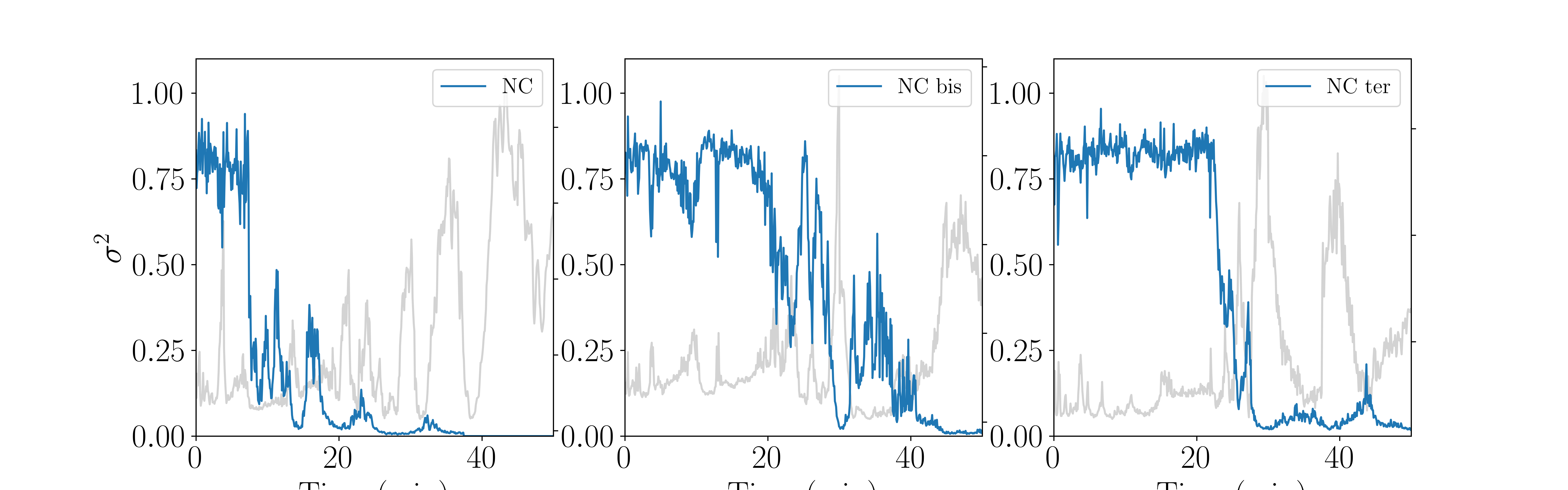

Supplement: Supplementary file 1 [file gels-11-00197-s001.zip › ESI-NCs.png]

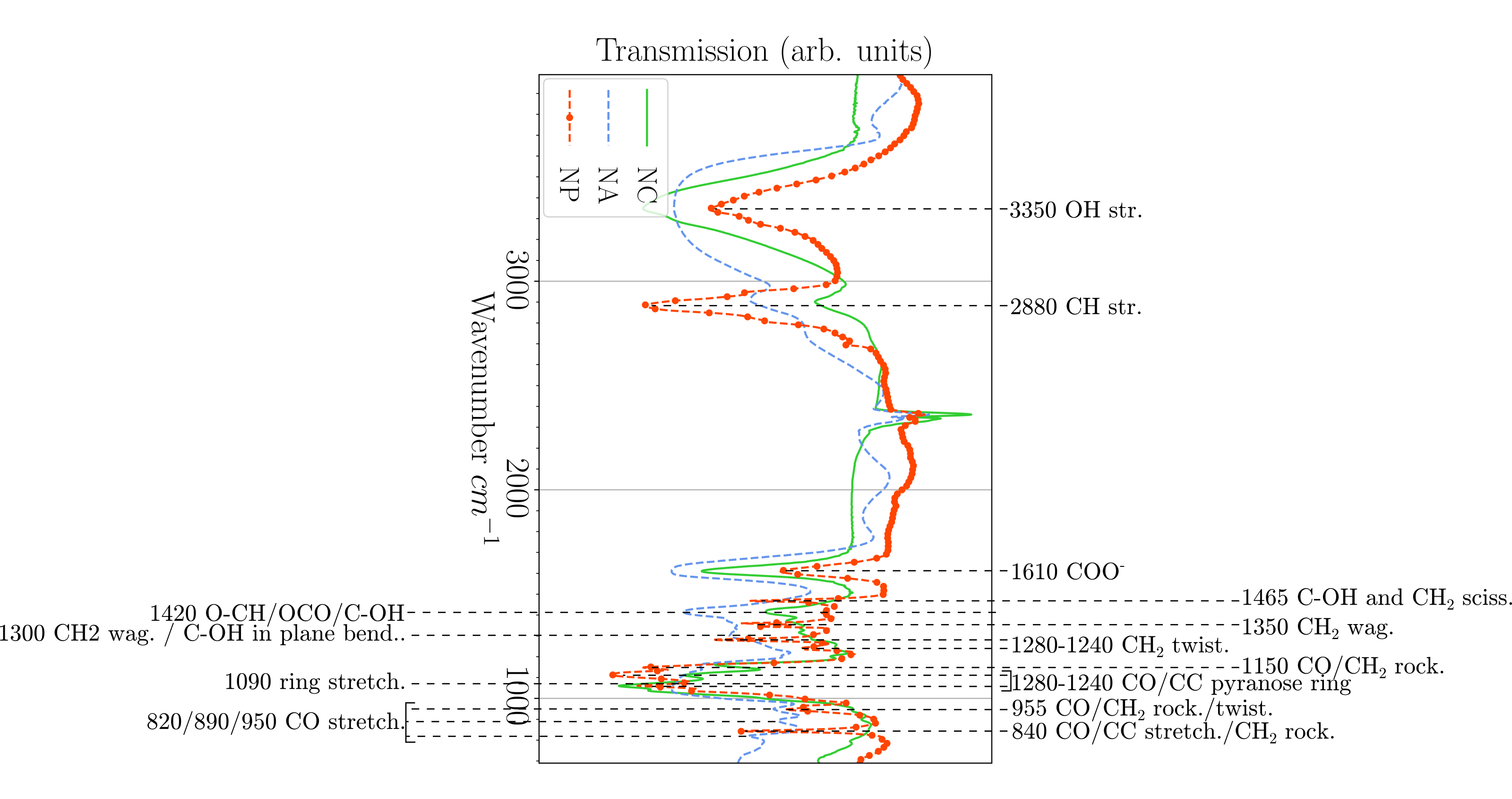

Supplement: Supplementary file 1 [file gels-11-00197-s001.zip › ESI_ftir2024_complete.png]

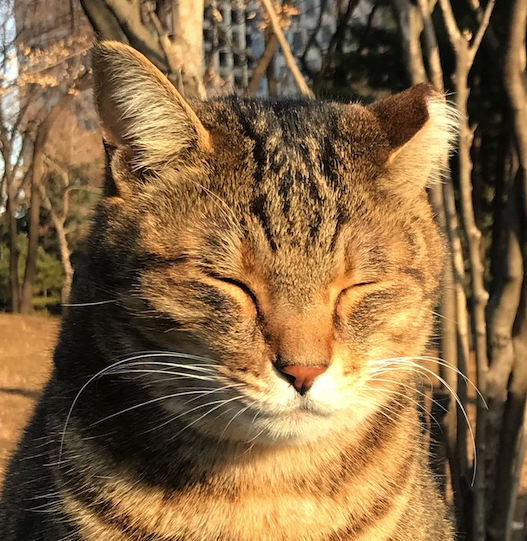

Supplement: Supplementary file 1 [file gels-11-00197-s001.zip › figures/cat_momo_1.png]

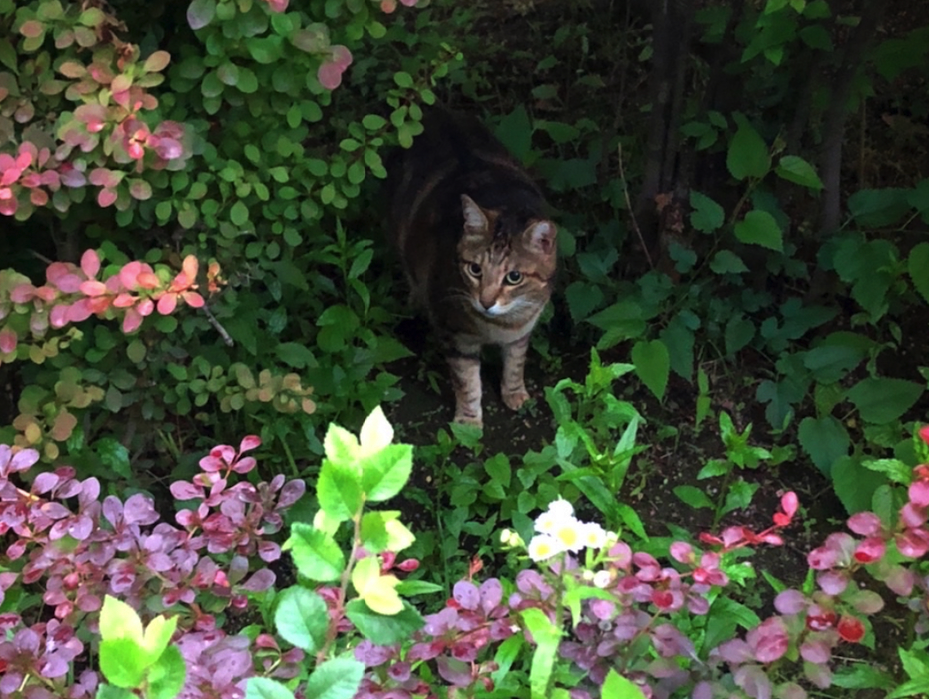

Supplement: Supplementary file 1 [file gels-11-00197-s001.zip › figures/cat_momo_2.png]

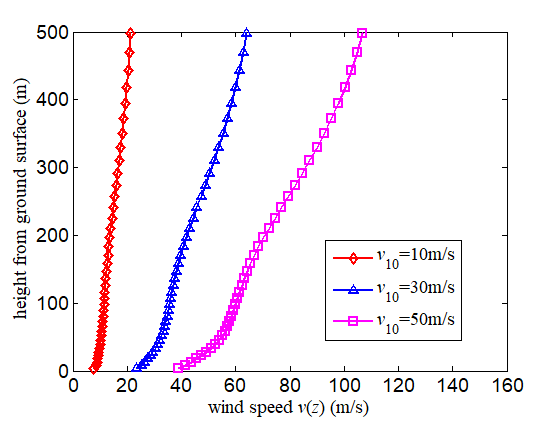

Supplement: Supplementary file 1 [file gels-11-00197-s001.zip › figures/fig_a.png]

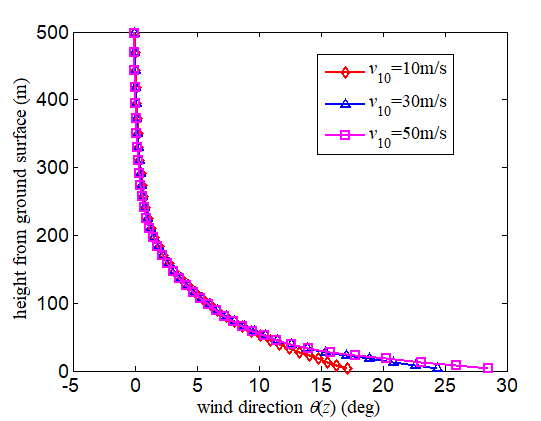

Supplement: Supplementary file 1 [file gels-11-00197-s001.zip › figures/fig_b.png]

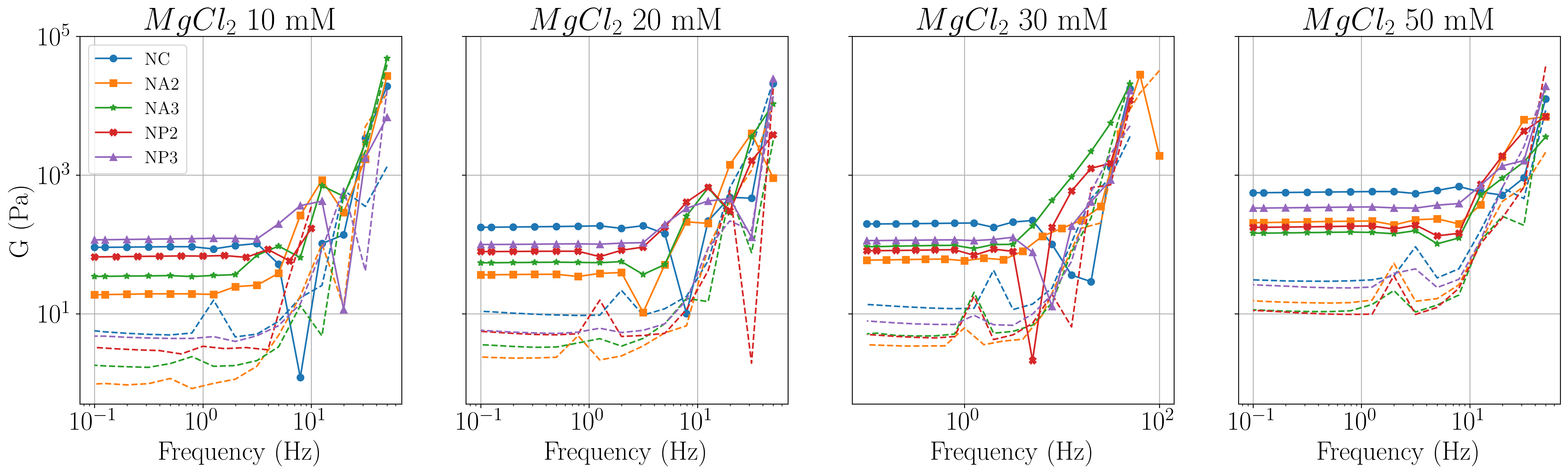

Supplement: Supplementary file 1 [file gels-11-00197-s001.zip › freq_sweep.png]

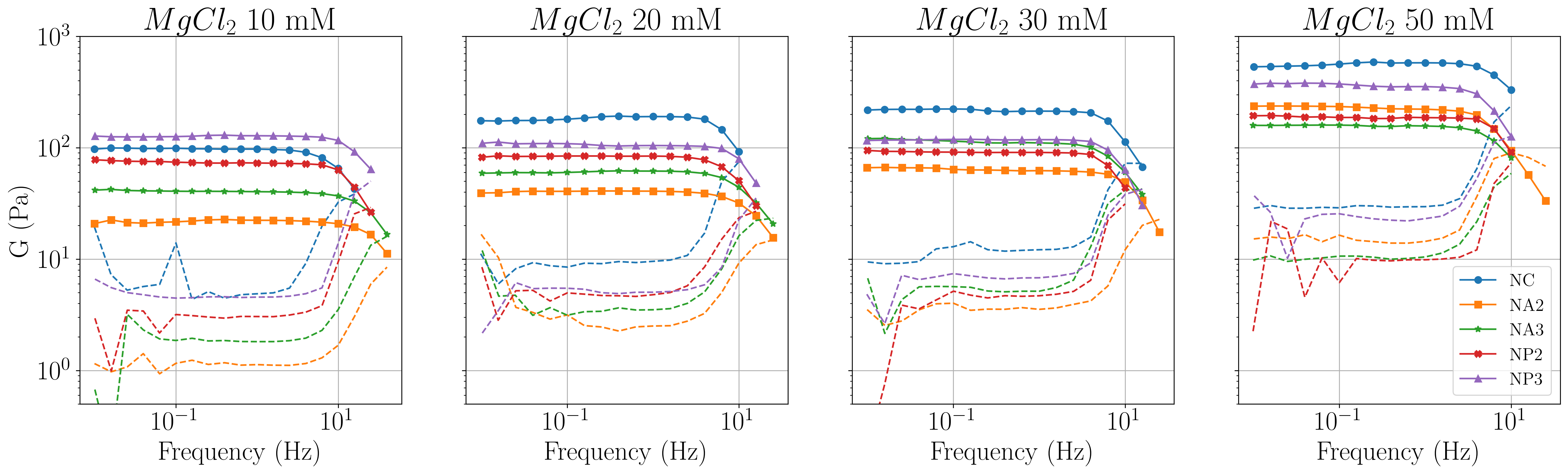

Supplement: Supplementary file 1 [file gels-11-00197-s001.zip › lver.png]
